# Coherent phonon control via electron-lattice interaction in ferromagnetic Co/Pt multilayers

**DOI:** 10.1038/srep22054

**Published:** 2016-03-01

**Authors:** Chul Hoon Kim, Je-Ho Shim, Kyung Min Lee, Jong-Ryul Jeong, Dong-Hyun Kim, Dong Eon Kim

**Affiliations:** 1 Department of Physics & Center for Attosecond Science and Technology, POSTECH, Pohang 376-73, South Korea; 2 Max Planck Center for Attosecond Science, Max Planck POSTECH/KOREA Research Initiative, Pohang, 376-73, South Korea; 3 Department of Material Science and Engineering and Graduate School of Energy Science and Technology, Chungnam National University, Daejeon 305-764, South Korea; 4 Department of Physics, Chungbuk National University, Cheongju 361-763, South Korea

## Abstract

The manipulation of coherent phonons in condensed systems has attracted fundamental interest, particularly for its applications to future devices. We demonstrate that a coherent phonon in Co/Pt nano-multilayer can be quantitatively controlled via electron-lattice coupling, specifically by changing the multilayer repeat number. To that end, systematic measurement of the time-resolved reflectivity and magneto-optical Kerr effect in Co/Pt multilayers was performed. The coherent phonon frequency was observed to be shifted with the change of the multilayer repeat number. This shift could be clearly explained based on the two-temperature model. Detailed analysis indicated that the lattice heat capacity and electron-lattice coupling strength are linearly dependent on the repeat number of the periodic multilayer structures. Accessing the control of coherent phonons using nanostructures opens a new avenue for advanced phonon-engineering applications.

The development of future devices in many applications demands an understanding not only of the behavior of carriers, but also of their surroundings such as the lattice (or phonon). In this respect, the generation and control of coherent phonons has attracted considerable interest. Recently, coherent heat transportation via coherent acoustic phonons has been demonstrated in superlattice multilayer structures^1^,^2^,^3^. It has been also reported that collective excitation of the phonon modes in multilayer samples induces a coherent phonon oscillation by impulsive stimulated Raman or Brillouin scattering^4^,^5^. Under conditions wherein sample thickness is well controlled, coherent acoustic phonons can be generated exclusively via stimulated Brillouin scattering, which affect the heat-transporting property of the multilayer structures. Luckyanova *et al.* reported the role of coherent acoustic phonons for thermal conduction in GaAs/AlAs semiconductor superlattice multilayer systems^1^. Their conductivity measurement with different multilayer repeat numbers revealed that coherent thermal conduction could occur via coherent phonon mediation through whole multilayer structures. Ravichandran *et al.* reported the crossover of phonon scattering from incoherent to coherent processes in high-quality perovskite superlattices of SrTiO_3_/CaTiO_3_ and SrTiO_3_/BaTiO_3_^2^. Ge *et al.* found that for thin MoS_2_ of less than 150 layers, the excited phonon frequency is linearly dependent on the sample thickness, whereas for thick samples, it does not change with thickness^3^.

All of these studies showed that the repeat number of multilayer structures is one of the control parameters for the engineering of their physical properties, particularly the coherent phonon behavior. In the present study, we undertook to understand the effect of the repeat number on the properties of coherent phonons in ferromagnetic multilayer systems. Compared to the semiconductor multilayers, unfortunately, little is known of the existence of coherent phonons in metallic multilayers^6^. Moreover, for ferromagnetic metallic multilayers, it has been known that there exists a spin precession excited by light or current pulses, whereas no experimental evidence has been provided for the existence of the coherent phonon in such systems. A ferromagnetic multilayer system such as a Co/Pt and Co/Pd multilayer could be interesting for its perpendicular magnetic anisotropy arising from strong spin-orbit coupling^7^ which can lead to significant spin-phonon interaction on an ultrafast time scale. A number of investigators have endeavored to understand the ultrafast photo-induced demagnetization process^8^,^9^; the role of the coherent phonon therein, or even its existence, however, remains unclear. To the best of our knowledge, there has been no detailed experimental study on the coherent phonon in a metallic ferromagnetic multilayer system that addresses the interaction dynamics among the coherent phonon, electron, and spin. Rather, these remain a scientific challenge to date.

In this paper, we report that coherent phonon in Co/Pt nano-multilayer can be quantitatively controlled via electron-lattice coupling. Using multilayer samples of different repeat numbers, it was found that a coherent phonon oscillation at a frequency of up to 100 GHz survives during the initial several tens of picoseconds in time-resolved reflectivity measurement and shifts gradually with varing the repeat number. We have also observed a similar effect in mageto-optical Kerr effect (MOKE) experiment. Based on detailed numerical analysis, we further concluded that such phonon oscillation behavior can be engineered via the change of electron-phonon coupling strength, which is effected by control of the multilayer repeat number.

## Results

We utilized time-resolved reflectivity and MOKE measurements (see Experimental section below for more detail) to examine the coherent phonon of Co/Pt multilayers. The light source employed was a commercial femtosecond multipass amplifier operating at a 780 nm wavelength, a 3 kHz repetition rate and a 25 fs pulse width. The pump fluence was adjusted to ~6.6 mJ/cm^2^ to avoid any photo-damage. A typical geometry comprising a half wave-plate, a Wollaston polarizer and two photodiodes was set up to record the transient Kerr signal. The angle of the magnet relative to the pump beam was set to 67°. The time resolution from the auto-correlation measurement was about 200 fs due to the pulse broadening coming from material dispersion and to the phase front tilting coming from the anlge between the pump and probe beam. Co/Pt multilayer samples on Si substrate were prepared under ambient temperature by means of DC magnetron sputtering. X-ray reflectivity measurement shows the excellent multilayer interface structure (Fig. S1 in supporting information). All the multilayer films are found to exhibit the perpendicular magnetic anisotropy out of the film plane confirmed by the vibrating sample magnetometer measurement, as shown in Fig. S1.

### Observation of phonon oscillation in Co/Pt multilayer

**Figure 1:**
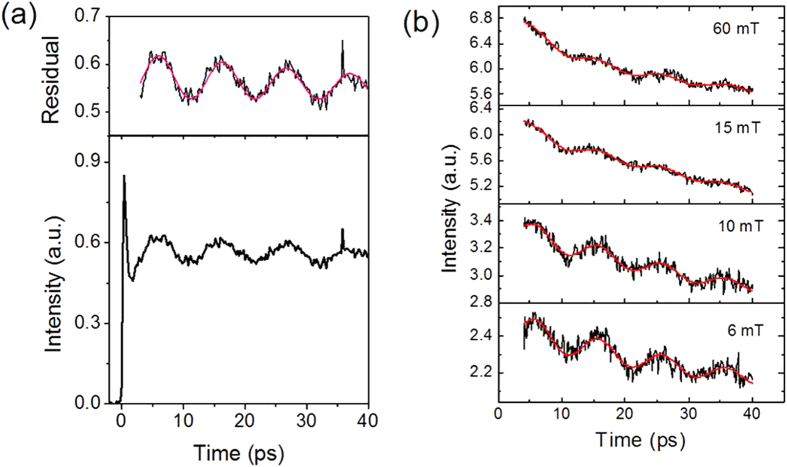
(**a**) Time-resolved reflectance under no external field for (6.3-Å Co/ 9.3-Å Pt)_10_ multilayer. The upper panel shows an LP-SVD fitting result (red line) of the residual of the reflectance signal. (**b**) Time-resolved MOKE under various external magnetic fields. Their LP-SVD fitting results (red line).

First, we examined the (6.3-Å Co/ 9.3-Å Pt)_10_ multilayer for its phonon and spin precession dynamics. Time-resolved reflectance measurement was employed to observe an optical response such as phonon dynamics, exclusively. Figure 1(a) shows the time-resolved reflectance with no external field where an oscillation was observed. The linear prediction singular value decomposition (LP-SVD) method was employed to fit the reflectance signal for extraction of exponentially damped sinusoids, using the equation


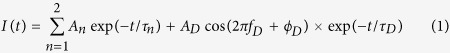


where *A*_*D*_, *f*_*D*_, *ϕ*_*D*_, and *τ*_*D*_ indicate amplitude, frequency, phase, and damping time, respectively^10^. The upper panel of Fig. 1(a) shows that the oscillation in the residual signal (minus the exponential decay) of the reflectance is well fit with *A*_*D*_ = 0.09, *f*_*D*_ = 99.5 GHz, *τ*_*D*_ = 38.5 ps, and *ϕ*_*D*_ = 141°. The frequency of that oscillation is about 99 GHz.

Figure 1(b) shows time-resolved (polar) MOKE signals of the (6.3-Å Co/ 9.3-Å Pt)_10_ film under external magnetic fields. In all of the signals, a coherent oscillation of about 99 GHz is apparent. Note that the oscillation frequency is independent of the magnetic field and the amplitude of the oscillation is also nearly insensitive to that (see Fig. S2 in supporting information). We carried out time-resolved MOKE experiments further on longer time scales up to 500 ps. The detailed analysis of these data shows that there exists a spin precessoin at a few GHz (Fig. S3 in supporting information). All of these facts imply that the observed oscillation around 99 GHz is not of magnetic origin. It is well known that MOKE signal contains optical artifacts within a few tens picosecond range^9^,^11^. Because time-resolved reflectivity measurements of various hetero-structures have shown that coherent phonons can be generated in multilayer film^12^,^13^,^14^, we are led to conclude that the observed oscillation originates from phonon dynamics that follow femtosecond optical excitation.

In summary, coherent phonon oscillation of up to 100 GHz survives only during the initial several tens of picoseconds; however, the spin precession, of only a few GHz, survives longer. This large frequency difference allowed us to investigate the coherent phonon dynamics and spin dynamics separately.

### Shift of phonon frequency in Co/Pt multilayers with respect to repeat number

**Figure 2:**
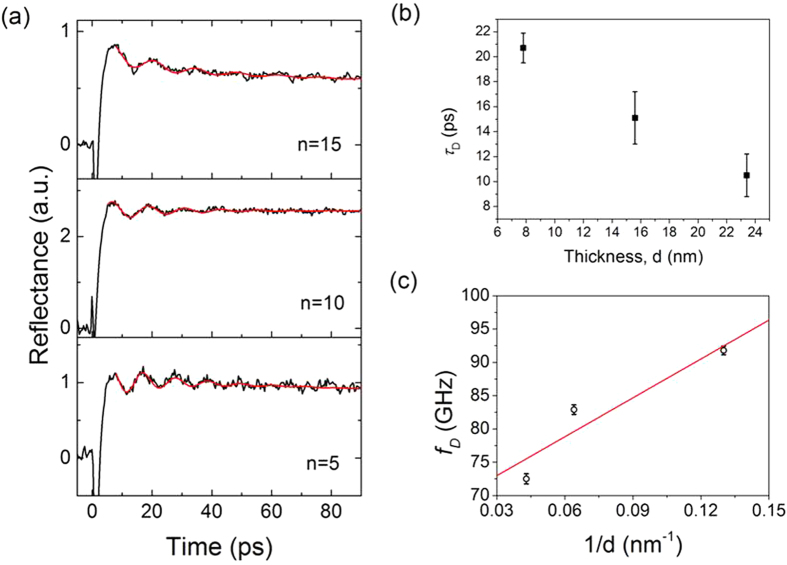
(**a**) Time-resolved reflectance signals for (6.3-Å Co/ 9.3-Å Pt)_n_ samples with n = 5, 10, 15. The red lines are LP-SVD fittings to the data. (**b**) Dependence of phonon oscillation and dephasing time on thickness, *d*. (**c**) Dependence of oscillation frequency on 1/*d* (circle). The error bars were taken from three independent measurements. The red line is a linear fit. The error bar is small enough that one may conclude that the oscillation frequency deviates from a linear dependence on 1/d.

We further examined the effect of the multilayer repeat numbers on coherent phonon dynamics using multilayer samples (6.3-Å Co/ 9.3-Å Pt)_n_ with the repeat numbers n = 5, 10, and 15. We observed the phonon oscillation behavior in a series of time-resolved reflectance measurements. Figure 2(a) shows the time-resolved reflectance signals along with the LP-SVD fitting results. Fitting parameters are listed in Table 1. In all cases, a phonon oscillation (70–90 GHz) was observed during the initial 80 ps. Note that the dephasing time (*t*_*D*_) also decrease as the number of layers increases (Fig. 2(b)).

A coherent phonon oscillation of 160 GHz in a 13-nm thick Pt metallic film on Si substrate has been reported^15^, showing increasing phonon frequency with decreasing film thickness and demonstrating, thereby, the thickness dependence of phonon oscillation. If the current phonon oscillation were due to the Pt layer in our samples, the phonon frequency should have been significantly greater than 160 GHz. The phonon oscillation of the Pt layer itself cannot explain the current observation.

The generation of coherent phonon oscillation may in fact be ascribed to the propagation of the coherent acoustic phonon wave-packet in a stacked film, which is to say that the sample acts as a Fabry-Perot interferometer. Wang *et al.* reported a similar experimental result wherein the coherent acoustic phonon wave-packet was generated in a semiconducting InMnAs/GaSb bilayer hetero-structure^13^. If the Co/Pt multilayer can be treated as one homogeneous medium, the oscillation frequency can be determined by the round-trip motion of the acoustic wave-packet through the whole structure. The frequency *f* is given by


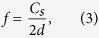


where *C*_*s*_ is the speed of sound in a material and *d* the total sample thickness. If *C*_*s*_ is constant, *f* would be proportional to 1/*d*. Figure 2(c) indicates that oscillation frequency, *f*, is not linearly proportional to 1/*d*. The error bar is small enough to support that the phonon frequency deviates from a linear dependence on 1/d . This implies that an interaction between lattice and electron (and/or spin) might play a role, resulting in the deviation from the 1/*d* dependence.

**Figure 3:**
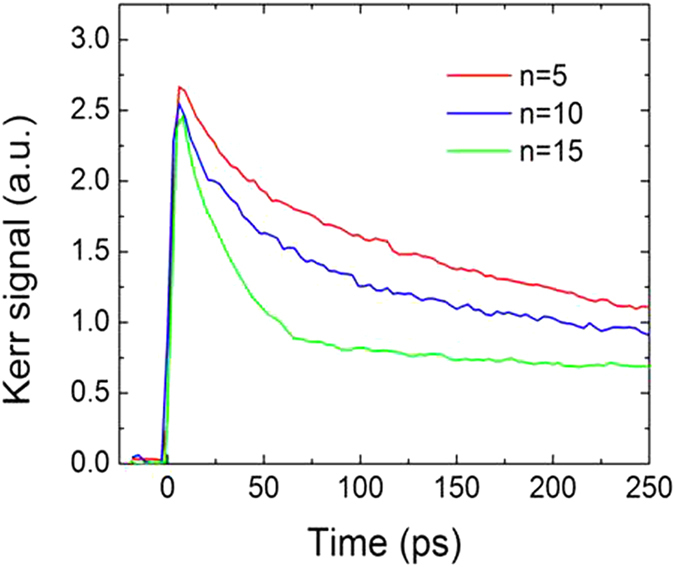
Time-resolved MOKE signals of [6.3-Å Co/ 9.3-Å Pt]_n_ films with n = 5, 10, and 15 under external magnetic field strength of 150 mT.

An epitaxial superlattice structure with lattice mismatch strains might cause a change of phonon oscillation, as reported in GaAs-In_x_Al_1-x_As strained-layer superlattices^16^ and ZnSeZnTe strained superlattices^17^ grown by molecular beam epitaxy. The Co/Pt multilayers in this present study, however, were grown by means of DC sputtering, which cannot produce an epitaxial layer on a substrate. Hence, we would expect a rather polycrystalline structure on each layer, implying that the lattice mismatch cannot be the cause of the observed phonon frequency shift. This lead us to an investigation of electron-lattice coupling. Since the effective relaxation time of the MOKE signal is known to be closely related to the coupling strength between electrons and lattices^18^,^19^, time–resolved MOKE measurements were carefully performed to study the dynamic behavior of electron /lattice coupling in Co/Pt multilayers. The time-resolved MOKE signals are plotted in Fig. 3 which shows that the initial relaxation time is smaller for larger n, even though the initial peak MOKE signals (at 5 ps) are almost indentical in all the samples. Schellekens *et al.* reported that the initial demagnetization (or intial peak MOKE signal) and subsequent remagnetization depends on the thickness of ferromagnetic film because of the different amount of absorption^20^. We note that in their work, the demagnetization (or intial peak MOKE signal) were different for different thikcnesses becasue of differetn strength of absoption. In our work, however, we observed the negligible changes of the initial peak MOKE signal at 5 ps for different n, which means that the absorption is almost identical in all the samples. Further numerical simulations were carried out, using the two temperature model for different source strengths (or equivalently, different absorptions) (see Fig. S4 in Supporting Information). The initial peak MOKE signals were proportional to the source strength. Interestingly, the simulation shows that the remagnetization becomes indeed slower but its variation is negligible in the condition of our study. Considering this simulation results and that the intial peak MOKE signals do not change for different repeated number (n), we may conclude that the skin-depth of the Co/Pt multilayers may be similar to or smaller than the thickness of (6.3-Å Co/ 9.3-Å Pt)_5_ and the coupling strength is indeed dependent on the repeat number.

On the other hands, we also observed the thickness dependence of the dephasing time (Fig. 2(b)). Ogi *et al.* showed that phonon oscillation from Pt layer can be modulated by its thickness and that the dephasing time was proportional to Pt thickness^15^. In this work, however, Pt layer thickness is kept constant and only the repeat number was controlled. Hence, the origin of the relaxation in our study should be different from that of a single Pt film itself. Possible mechanisms for the observed thickness dependence of the phonon damping time are (1) a scattering from the interfaces between Pt and Co layers because mixing of atoms and presence of dislocations at interfaces causes the interface scattering^21^ and/or (2) the coupling strength change between electron and phonon because a strong coupling interaction causes a faster relaxation in phonon oscillation. In any case, this observation also leads us to the investigation of the coupling interaction.

## Discussion

### Quantitative analysis of time-resolved remanent magnetizations of Co/Pt multilayer

**Figure 4:**
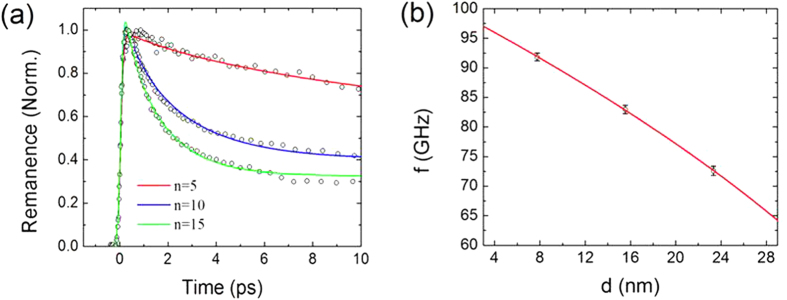
(**a**) Time-resolved remanent magnetization for (6.3-Å Co/ 9.3-Å Pt)_n_ samples with n = 5, n = 10, and n = 15 (circles) and their global fitting results (solid lines). (**b**) Fitting of coherent phonon frequency (as shown in Fig. 2(c)), based on function of 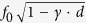, where *γ* is constant and *d* is total thickness of multilayer sample.

For the purposes of a quantitative analysis of the electron-lattice coupling in demagnetization dynamics, we measured the time-resolved remanent magnetization (i.e., the magnetization at a zero external magnetic field)^8^,^22^. At each time delay of time-resolved MOKE measurement, a hysteresis loop measurement was performed, from which the temporal changes of remanent magnetization were obtained. Figure 4 shows the temporal behavior of remanent magnetization signal (indicated by the open circles) for the different repeat numbers n = 5, 10 and 15. We observe that the initial relaxation during the first 10 ps is dramatically different for the respective repeat numbers. The fast relaxation process after photo-induced demagnetization can be attributed to the interaction dynamics between the heat reservoir of electron, phonon and spin. This lead us to assume that the interaction dynamics between heat reservoirs can also be varied according to the repeat number, which also causes the phonon frequency shift.

At the outset, we applied a three-temperature model^23^ the results of which showed, however, almost no difference between the spin and the electron temperature, as has been widely assumed for ferromagnetic metal systems. Thus, we then analyzed the time-resolved remanent magnetization by means of a two-temperature model^8^,^22^. We considered only the lattice and electron temperatures using the equations


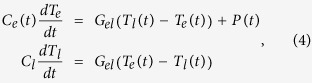


where *T*_*e*_ and *T*_*l*_ are the electron and lattice temperature, *C*_*e*_ and *C*_*l*_ are electron- and lattice-specific heat, *G*_*el*_ represents an electron-lattice coupling that corresponds to electron-phonon interaction, *P*(*t*) accounts for the heating by laser pulse (pulse duration on the sample is expected to be 50 fs, due to dispersive broadening of original pulse through various optical elements), and *C*_*e*_(*t*) is assumed to be linearly proportional to *T*_*e*_ for all samples.

Time-resolved remanent magnetization *M*(*T*_*e*_(*t*)) can be calculated as^22^


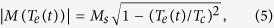


where *M*_*s*_ is the spin magnetization, and *T*_*c*_ the Curie temperature. It is known that the Curie temperature of cobalt-based ferromagnetic thin films varies according to thickness^24^,^25^,^26^. P. Bruno reported, for example, that *T*_*c*_ is dramatically decreased for films of 5 or fewer atomic layers, whereas it remains almost constant for films of more than 10 atomic layers^26^. For 5-atomic-layer samples, the ratio of *T*_*c*_ between the thin film and the thick film was not unity, but about 0.8, allowing an assumption that the effect due to the Curie temperature change by thickness difference in our Co/Pt multilayer samples is minor. That is, it was indicated that *T*_*c*_ is constant (1645 K for Cobalt) for all such samples.

In order to quantitatively characterize the major effects of *P*(*t*), *C*_*e*_, *G*_*el*_ and *C*_*l*_ on *M*(*T*_*e*_(*t*)) in the two-temperature model, respectively (see Supporting Information for more details), a series of numerical analyses was performed. The results suggest the following parameter changes in accordance to the periodic sample structures:


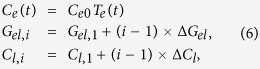


where *i* = 1, 2, and 3 correspond to super-lattice samples with 5, 10, and 15 bi-layers, respectively, and Δ*G*_*el*_ is the effective size of the change due to a set of 5 multilayers. *C*_*l*_ is assumed not to be a function of the lattice temperature, since it has only a weak temperature dependence at high temperatures. We used the two-temperature model to globally fit all of the remanent magnetization with the constraints given in Eq. (6).

The global fitting results are shown as solid lines in Fig. 4. Fitting parameters are listed in Table 2. The global fitting to time-resolved remanent magnetization for all the samples was quite good, indicating that the constraints given in Eq. (6) are valid. This result further implies that both the interaction coupling *G*_*el*_ and the lattice-specific heat *C*_*l*_ increase linearly with the multilayer repeat number.

### Coherent phonon frequency shift via electron-phonon interaction

We also attempted to fit the coherent phonon frequency shift with the empirical formula,


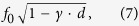


where *f*_0_ = 100 GHz, *γ* = 203 nm^−1^, and *d* is the total thickness of a multilayer, as shown in Fig. 4(b). The fitting is quite good. There have been several experimental studies on the effect of coupling strength on phonon oscillation. Kim *et al.* reported frequency shifts of the internal phonon modes in a polycrystalline superlattice sample^27^ where a strong electron-phonon coupling is attributed to frequency shifts. They showed that the high-temperature phonon frequency shift from the electron-phonon interaction can be approximated by


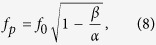


where *f*_0_ is an intrinsic coherent phonon frequency, *α* a polaron bandwidth, and *β* a time-independent parameter containing the contribution from the electron-phonon interaction strength^27^,^28^. Eq. (8) and the observation of good fitting to data by Eq. (7) indicate that the electron-phonon coupling strength may be proportional to the total thickness *d* or the repeat number, and further confirms the simple linear increase of *G*_*el*_ with respect to the multilayer repeat number. This linear increase of *G*_*el*_ and *C*_*l*_ implies that the coherent phonon oscillation behavior can be controlled via a modification of the electron-lattice interaction, which is to say, by changing the multilayer repeat number. Thus, the quantitative control of heat transfer via a coherent phonon, further advance from the work done by Luckyanova *et al.*^1^, might become possible on an ultrafast time scale.

In conclusion, we have experimentally discovered the existence of the coherent phonon in ferromagnetic metallic Co/Pt multilayers, where the lattice-electron coupling has been intensively investigated by time-resolved reflectivity and MOKE measurement. It was noted that the phonon frequency changes with respect to the multilayer repeat number. A detailed quantitative analysis of time-resolved remanent magnetization showed that the electron-lattice coupling strength varies according to the multilayer repeat number. This served to demonstrate that electron-lattice coupling can be controlled by changing the multilayer repeat number, leading to a possible practical engineering of the coherent phonon behavior responsible for the ultrafast heat transfer in nanoscale devices.

## Experimental Section

Two sets of thin Co/Pt multilayer samples on Si substrate were prepared at an ambient temperature by means of DC magnetron sputtering. One sample was (6.3-Å Co/ 9.3-Å Pt)_10_, wherein the thicknesses of the Co and Pt sub-layers were fixed to 6.3 Å and 9.3 Å, respectively. The other samples were (6.3-Å Co/ 9.3-Å Pt)_n_, wherein the multilayer repeat number, n, was varied from n = 5 to 10 and to 15. The former sample was used to show the co-existence of both phonons and spin precession in a MOKE signal, whereas the latter samples were used to investigate the effect of the repeat number on phonon dynamics, exclusively.

We utilized time-resolved pump-probe methods to examine the coherent phonon and spin precession dynamics of the Co/Pt multilayers. The light source was a commercial CEP (Carrier Envelope Phase) stabilized multipass amplifier (Femtopower, Femtolaser. Inc.) operating at a 780 nm wavelength a 3 kHz repetition rate and a 25 fs pulse width. A pair of BK7 prims was used to adjust the group velocity dispersion of the amplifier output. A pellicle beam splitter (50:50) was used to split the output to obtain pump and probe beams. Both beams were set up for s-polarizations. The intensities of both beams were adjusted by means of a half wave-plate together with a linear polarizer. The pump beam was focused on the sample at a normal incidence. The mixing angle between the pump and the probe beam was set to 35°. The angle of the magnetic field with respect to the direction of the pump beam was set to 67°. The pulse energy of the pump beam was adjusted to approximately 6.6 mJ/cm^2^. The pulse width on sample was broadended to 50 fs because of higher order dispersions of various optics. Probe pulses reflected from the sample surface were sent to the Wollaston polarizer to detect the s- and p- polarized outputs using two Si photo-diodes. A chopper was used to modulate the pump. A lock-in amplifier was employed to measure the pump-modulated probe intensities. For reflectance measurement, the reflected probe beam was directly measured to record its intensity change. For time-resolved remanent magnetization measurement, we measured a pump-induced time-resolved hysteresis loop at each time delay, and then extracted the time-resolved Kerr signals at a given magnetic field strength. The time resolution, measured by the auto-correlation measurement, was about 200 fs, due to the phase-front tilt caused by the 35° mixing angle between the pump and the probe beam. To minimize any artifacts from the long term drift in the laser output, we made multiple measurements (more than 50 times depending on S/N ratio) with the scanning time of 5 minutes and got averaged signals.

## Additional Information

**How to cite this article**: Kim, C. H. *et al.* Coherent phonon control via electron-lattice interaction in ferromagnetic Co/Pt multilayers. *Sci. Rep.* **6**, 22054; doi: 10.1038/srep22054 (2016).

## Supplementary Material

Supplementary Information

## Figures and Tables

**Table 1:** LP-SVD fitting results for time-resolved reflectance signals of (6.2-Å Co/ 8-Å Pt)_n_.

n	d (nm)	1/d (nm^−1^)	*A*	*τ*_*D*_ (ps)	*f*_*D*_(GHz)	*ϕ*_*D*_ (deg.)
5	7.8	0.13	0.118 ± 0.05	20.7 ± 1.2	91.8 ± 0.65	3.91 ± 1.1
10	15.6	0.064	0.149 ± 0.08	15.1 ± 2.1	82.9 ± 0.71	4.13 ± 1.5
15	23.4	0.043	0.128 ± 0.07	10.5 ± 1.7	72.5 ± 0.78	4.04 ± 1.3

Note that the fitting errors were taken from three independent measurements.

**Table 2:** Global fitting results for time-resolved remanent magnetization signals.

*C*_*e0*_(Jm^−3^K^−2^)	*G*_*el,1*_(Wm^−3^K^−1^)	*ΔG*_*el*_	*C*_*l,1*_(Jm^−3^K^−1^)	*ΔC*_*l*_
2.09 × 10^3^	3.11 × 10^16^	3.13 × 10^17^	7.66 × 10^5^	7.14 × 10^5^

## References

[b1] Luckyanova M. N. *et al.* Coherent Phonon Heat Conduction in Superlattices. Science 338, 936 (2012). 23161996 10.1126/science.1225549

[b2] Ravichandran J. *et al.* Crossover from incoherent to coherent phonon scattering in epitaxial oxide superlattices. Nat. Mater. 13, 168 (2014). 24317186 10.1038/nmat3826

[b3] Ge S. F. *et al.* Coherent Longitudinal Acoustic Phonon Approaching THz Frequency in Multilayer Molybdenum Disulphide. Sci. Rep. 4, 5722 (2014). 25031087 10.1038/srep05722 PMC4101472

[b4] Cheng L.-T. & Nelson K. A. Ferroelectric phase transition in RbH_2_PO_4_: Picoseond time-resolved impulsive stimulated Brillouin scattering experiments. Phys. Rev. B 37, 3603 (1988).

[b5] Damen E. P. N., Arts A. F. M. & Dewijn H. W. High-Frequency Monochromatic Acoustic-Waves Generated by Laser-Induced Thermomodulation. Phys. Rev. Lett. 74, 4249 (1995). 10058453 10.1103/PhysRevLett.74.4249

[b6] Dieleman D. L., Koenderink A. F., van Veghel M. G. A., Arts A. F. M. & de Wijn H. W. Transmission of coherent phonons through a metallic multilayer. Phys. Rev. B 64, 174304 (2001).

[b7] Nakajima N. *et al.* Perpendicular magnetic anisotropy caused by interfacial hybridization via enhanced orbital moment in Co/Pt multilayers: Magnetic circular x-ray dichroism study. Phys. Rev. Lett. 81, 5229 (1998).

[b8] Beaurepaire E., Merle J.-C., Daunois A. & Bigot J.-Y. Ultrafast Spin Dynamics in Ferromagnetic Nickel. Phys. Rev. Lett. 76, 4250 (1996). 10061239 10.1103/PhysRevLett.76.4250

[b9] Koopmans B., Ruigrok J. J. M., Dalla Longa F. & Jonge W. J. M. Unifying Ultrafast Magnetization Dynamics. Phys. Rev. Lett. 95, 267207 (2005). 16486397 10.1103/PhysRevLett.95.267207

[b10] Barkhuijsen H., Beer R. D., Bovee W. M. M. J. & Ormondt D. V. Retrieval of Frequencies, Amplitudes, Damping Factors, and Phases from Time-Domain Signals Using a Linear Least-Squares Procedure. Journal of Magnetic Resonance 61, 465 (1985).

[b11] Wang J. In Optical Techniques for solid-state materials characterization Ch. 13, 467 (Taylor & Francis Group, LCC, 2012).

[b12] Noe G. T. *et al.* Superlattice Microst 52, 1071–1077 (2012).

[b13] Wang J. *et al.* Propagating coherent acoustic phonon wave packets in In_x_Mn_1-x_As/GaSb. Phys. Rev. B 72, 153311 (2005).

[b14] Makarona E. *et al.* Coherent generation of 100 GHz acoustic phonons by dynamic screening of piezoelectric fields in AlGaN/GaN multilayers. Appl. Phys. Lett. 81, 2791 (2002).

[b15] Ogi H., Fujii M., Nakamura N., Shagawa T. & Hirao M. Resonance acoustic-phonon spectroscopy for studying elasticity of ultrathin films. Appl. Phys. Lett. 90, 191906 (2007).

[b16] Nakayama M. *et al.* Raman study of GaAsIn_x_A_l1x_As strainedlayer superlattices. J Appl. Phys. 58, 4342 (1985).

[b17] Kobayashi M., Konagai M., Takahashi K. & Urabe K. Lattice strain and lattice dynamics of ZnSeZnTe strainedlayer superlattices. J. Appl. Phys. 61, 1015 (1987).

[b18] Weber M. C., Hillebrands B., Moshnyaga V. & Samwer K. Spin-lattice relaxation phenomena in manganite La_0.7_Sr_0.3_MnO_3_ thin films. Europhys. Lett. 73, 285 (2006).

[b19] Laraoui A. *et al.* Study of individual ferromagnetic disks with femtosecond optical pulses. J. Appl. Phys. 101, 09C105 (2007).

[b20] Schellekens A. J., Verhoeven W., Vader T. N. & Koopmans B. Investigating the contribution of superdiffusive transport to ultrafast demagnetization of ferromagnetic thin films. Appl Phys Lett 102, 252408 (2013).

[b21] Hepplestone S. P. & Srivastava G. P. Theory of interface scattering of phonons in superlattices Phys. Rev. B 82, 144303 (2010).

[b22] Malinowski G. *et al.* Control of speed and efficiency of ultrafast demagnetization by direct transfer of spin angular momentum. Nat Phys 4, 855 (2008).

[b23] Bigot J. Y., Vomir M., Andrade L. H. F. & Beaurepaire E. Ultrafast magnetization dynamics in ferromagnetic cobalt: The role of the anisotropy. Chem Phys 318, 137 (2005). 10.1103/PhysRevLett.94.237601 16090502

[b24] Jensen P. J., Dreyssé H. & Bennemann K. H. Thickness dependence of the magnetization and the Curie temperature of ferromagnetic thin films. Surface Science 269/270, 627 (1992).

[b25] Rausch R. & Nolting W. The Curie temperature of thin ferromagnetic films. J. Phys. Condens. Mat. 21, 376002 (2009). 10.1088/0953-8984/21/37/376002 21832358

[b26] Bruno P. Theory of the Curie temeprature of cobalt-based ferromagnetic ultrathin films and multilayers. J. Magn. Soc. Jpn. 15, 15 (1991).

[b27] Kim K. H., Gu J. Y., Choi H. S., Park G. W. & Noh T. W. Frequency shifts of the internal phonon modes in La_0.7_Ca_0.3_MnO_3_. Phys Rev Lett 77, 1877 (1996). 10063194 10.1103/PhysRevLett.77.1877

[b28] Mahan G. D. Many-Particle Physics. (Plenum, 1990).

