# Supplementary material for: Coherent phonon control via electron-lattice interaction in ferromagnetic Co/Pt multilayers
Source: Sci Rep. 2016 Mar 1;6:22054. doi: 10.1038/srep22054 (PMC4771999; doi:10.1038/srep22054)
Supplement: Supplementary Information [file srep22054-s1.pdf]

Supporting Information

Coherent phonon control via electron-lattice interaction in ferromagnetic Co/Pt multilayers

Chul Hoon Kim,[1,2] Je-Ho Shim,[3] Kyung Min Lee,[4] Jong-Ryul Jeong,[4] Dong-Hyun Kim,[3*] and Dong Eon Kim[1,2+]

1. Department of Physics, Center for Attosecond Science and Technology, POSTECH, Pohang, Kyungbuk 790-784, South Korea
2. Max Planck Center for Attosecond Science, Pohang, Kyungbuk 790-784, South Korea
3. Department of Physics, Chungbuk National University, Cheongju, Chungbuk 361-763, South Korea
4. Department of Material Science and Engineering and Graduate School of Green Energy Technology, Chungnam National University, Daejeon 305-764, South Korea

(1) Co/Pt multilayers structures and magnetic property

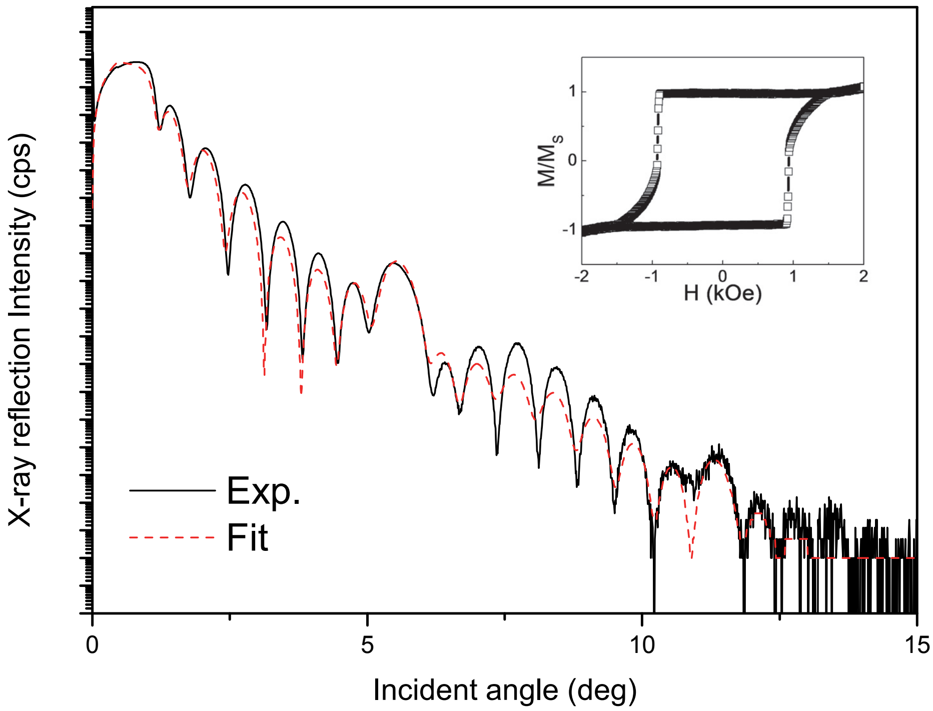

Figure S1. X-ray reflection measurement (solid) and fitting curve (dash). The inset figure is the static hysteresis loop of (6.3-Å Co/ 9.3-Å Pt)$_5$.

The Co/Pt multilayer has been fabricated with well-defined interface structures, which are confirmed with the X-ray reflection measurement as shown in the figure for the case of n = 5. Minor peaks from the multilayer interfaces are clearly distinguishable, providing the best fitting result of t_{Co} = 6.3 Å and t_{Pt} = 9.3 Å. The corresponding static magnetic hysteresis loop for n = 5, measured by the vibrating sample magnetometer along the out-of-the plane of the sample is plotted as in the inset, clearly showing that the film has a perpendicular magnetic anisotropy It guarantees that each layer of the Co is ferromagnetically ordered along the easy axis out of the film plane. The perpendicular magnetic anisotropy is found in all the sample in the present study.

(2) Phonon oscillations resolved in the time-resolved MOKE

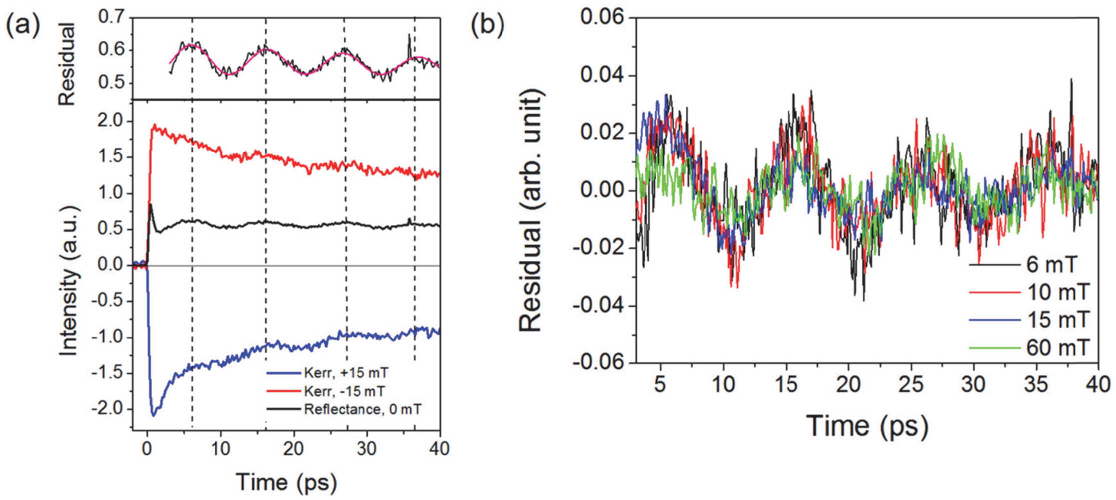

Figure S2. (a) Time-resolved MOKE under external bias fields of +/- 15 mT and time-resolved reflectance under no external field for (6.3-Å Co/ 9.3-Å Pt)$_5$. The upper panel shows a LP-SVD fitting result (red line), showing the variation of the residual of the reflectance signal. The dotted vertical lines are just guidelines for eye to examine the phase of oscillations in MOKE and reflectance data. (b) Residuals of the time-resolved MOKE under various external bias fields (Fig. 2(b)).

Generally, time-dependent MOKE signal, $\Delta I_M(t)$, is given by:[1]

$$\Delta I_M(t) = 2R_0\Delta\theta(t) + 2\Delta R(t)\theta_0 , \qquad \text{S(1)}$$

where R and θ correspond to reflectance and Kerr rotation, respectively. In many cases, the first term is larger than the second terms so that time-resolved MOKE directly probes magnetization dynamics; however, we need to consider the time-dependent change of reflectance $\Delta R(t)$ if the second term is not negligible.

We have observed the same coherent oscillation in both time-resolved MOKE and reflectivity measurments. The phase of the weak oscillations in the MOKE signals under +/- 15 mT is identical to that in the reflectance (see the vertical dotted lines in Fig. S2(a)).

Figure S2(b) shows the oscillating residuals of the MOKE signals, obtained by using the LP-SVD method. Note that the residual signal is an exponentially damped sinusoidal term (the 2nd term in Eq. (1) in the main text), where the exponential term by remagnetization was removed. Their oscillating features are independent of the external bias field, indicating that the phonon oscillation is insensitive to the magnetic field in the condition of our study.

All of these observations indicates that the observed oscillation was not from a magnetic response.

(3) Observation of spin precession in time-resolved MOKE

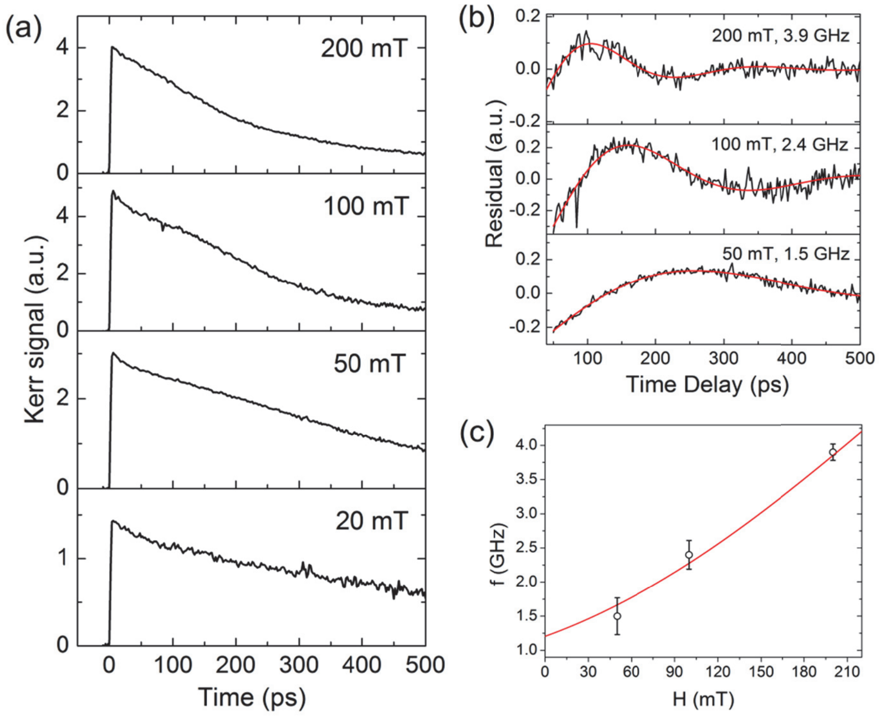

Figure S3. (a) Time-resolved MOKE signals under different field strengths for (6.3-Å Co/ 9.3-Å Pt)$_{10}$ film, (b) Residual signals with LP-SVD fittings (red lines), (c) Change of spin precession frequency with respect to field strength. The red line is a fitting using Eq. S(1).

We carried out time-resolved MOKE experiments on longer time scales up to 500 ps. Figure S3(a) shows the time-resolved MOKE signals for different strengths of magnetic field. Careful observation has indicated weak oscillations on top of the decay signals, the LP-SVD method was employed to fit the MOKE signals. These weak oscillations, as shown in Figure S3(b), became conspicuous when we subtracted the exponential components from the MOKE signals and considered only the residual signals. According to the behavior of residual signals, weak coherent oscillation at a few GHz appears as the magnetic field strength increases, and its frequency is also changed by the field strength.

The dependence of spin precession frequency f on the strength of a magnetic field[3] can be explained by

$$f = (\gamma / 2\pi)\sqrt{H_1 H_2} \ , \qquad \text{S(2)}$$

where $H_1 = H\cos(\theta_H - \theta) + H_k^{eff}\cos^2\theta$ and $H_2 = H\cos(\theta_H - \theta) + H_k^{eff}\cos 2\theta$, H_k^{eff} is effective perpendicular magnetic anisotropy field, γ the gyromagnetic ratio, H the external magnetic field, θ_H the angle of the magnetic field (in our case 67°), and θ the magnetization angle. The magnetization angle θ was calculated numerically according to the relation $\sin 2\theta = (2H / H_k^{eff})\sin(\theta_H - \theta)$ and the fitting parameters, γ and H_k^{eff}. Figure S3(c) shows the fitting results (red lines) using Eq. S(2). The experimental data well fit with $\gamma = 12.6\ \mathrm{Mrad/s \cdot Oe}$ and $H_k^{eff} = 0.56\ \mathrm{kOe}$. Thus, the low-frequency few-GHz-scale oscillation could be ascribed to the spin precession.

Hence, it is concluded that this oscillation is of magnetic origin, It is observed that spin precessional motion is in a frequency range of a few GHz. Indeed this is consistent with recent MOKE experimentation with Pt/8-Å Co/Pt film in Mizukami's work, where the precession frequency was reported to be lower than 10 GHz.[2]

We have further analyzed the damping of spin precessional motions. The effective damping constant α is achieved from the relation $\alpha = 1/2\pi f\tau_D$, where τ_D is determined from the analysis of spin precession signals (Figure S3(b)) by adopting Eq. (1). The best fit yields the damping parameter α of about 0.45±0.10. The value of α for the case of t_{Co} (thickness of cobalt layer) = 8 Å, measured by TR-MOKE in Mizukami's work, is about 0.25.[2] The t_{Co} in the present study is 6.3 Å, for which the α value of about 0.45 is expected according to the Mizukami's work. Relatively strong damping behavior in the present study is not exceptional, compared to the previous results in Pt/Co/Pt trilayer It has been known that α alpha value determined measured by the TR-MOKE is found to be substantially different from the values measured by ferromagnetic resonance although the origin of the discrepancy is still controversial.[3,4]

(4) Characteristic behavior of demagnetization with respect to change of source term and electron- and lattice-specific heat and electron-lattice interaction strength

The two-temperature model by Bigot *et al.* `has often been employed to quantitatively analyze the time-resolved MOKE signals

$$\begin{aligned} C_e(t)\frac{dT_e}{dt} &= G_{el}\left(T_l(t)-T_e(t)\right)+P(t) \\ C_l\frac{dT_l}{dt} &= G_{el}\left(T_e(t)-T_l(t)\right)-\kappa_l\vec{\nabla}^2 T_l\left(t,\vec{r}\right) \end{aligned}, \quad \text{S(1)}$$

where $T_e(t)$ and $T_l(t)$ are the time-dependent electron and lattice temperatures, respectively, $C_e(t)$ the temperature-dependent electron-specific heat, G_{el} the electron-lattice interaction strength, C_l the lattice-specific heat, and κ_l the thermal conductivity.

We performed a series of simulations to determine how the specific heat coefficient and the electron-lattice interaction strength would change electron and lattice temperature as well as magnetization. In our simulations, the thermal diffusion term was neglected due to the lateral uniformity, and we assumed that $C_e(t)$ is proportional to $T_e(t)$. Hence, Eq. S(1) can be simplified as

$$\begin{aligned} C_{e0}T_e(t)\frac{dT_e}{dt} &= G_{el}\left(T_l(t)-T_e(t)\right)+P(t) \\ C_l\frac{dT_l}{dt} &= G_{el}\left(T_e(t)-T_l(t)\right) \end{aligned}, \quad \text{S(2)}$$

where C_{e0} is the electron-specific heat coefficient.

The time-resolved remanence signal, $M(T_e(t))$, can be calculated using the equation

$$\left|M\left(T_e(t)\right)\right| = M_s\sqrt{1-\left(T_e(t)/T_c\right)^2}\,, \quad \text{S(3)}$$

where M_s is the spin magnetization and T_c is the Curie temperature (1645 K for Cobalt). We assume here that the electron temperature is the same as the spin temperature. In the present work, we also applied a three-temperature model, wherein almost no difference was found between the spin and the electron temperature; therefore, we simply adopted the two-temperature model to focus on coherent phonon behavior.

For practical convenience, the following equation was used to account for a real MOKE signal that is modulated by the pump beam to yield a zero background below time zero:

$$S_M(t) = A \times \left[M\left(T_e(t)\right) - M(300 \text{ K}) \right], \qquad \text{S(4)}$$

where A is an arbitrary amplitude parameter.

Numerical analysis was performed to determine the effects of the three parameters (C_{e0}, G_{el} and C_l) on $S_M(t)$. Figure S3 shows the results. In the simulation, $P(t)$ was a Gaussian function with $7\text{x}10^{21}$ W/m^3 and 200 fs width, and A was -1.

A. Effect of Source term, *P*(*t*)

Figure S4 shows the effect of source term on M while the other parameters were fixed; C_{e0}, G_{el} and C_l are $6\text{x}10^3$ $\text{Jm}^{-3}\text{K}^{-1}$, $6\text{x}10^{17}$ $\text{Wm}^{-3}\text{K}^{-1}$ and $3.5\text{x}10^6$ $\text{Jm}^{-3}\text{K}^{-1}$, respectively. The simulation result shows that the initial maximum amplitude of $S_M(t)$ was decreased as pump irradiance increased. Note that the initial relaxation time of SM(t) was also changed slightly.

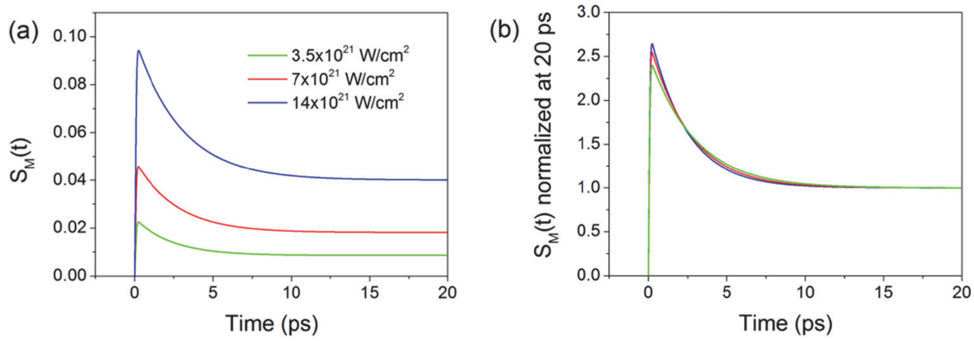

Figure S4. Numerical simulation results characterizing the effect of the source term, $P(t)$. (a) the amplitude of $S_M(t)$ increases as power increases. (b) the initial relaxation (remagnetization) of $S_M(t)$ increases slightly as power increases.

B. Effect of Electron-specific heat, C_e

Figure S5(a) shows the effect of C_{e0} on M while the other parameters were fixed; G_{el} and C_l are $6\text{x}10^{17}$ $\text{Wm}^{-3}\text{K}^{-1}$ and $3.5\text{x}10^6$ $\text{Jm}^{-3}\text{K}^{-1}$, respectively. The simulation result shows that the initial maximum amplitude of $S_M(t)$ was decreased as C_{e0} increased.

Figure 3 in the main text shows the experimental Kerr signals for all of the samples. Note that the initial maximum amplitude of the MOKE signals was almost the same in all cases. In connection with the simulation results, this implies that the electron-specific heat coefficient

is the same for all of the samples. Hence, we could assume that the C_e is not a function of the multilayer repeat number.

C. Effect of Coupling strength, G_{el}

Figure S5(b) shows the effect of G_{el} on T_e while C_{e0} and C_l were fixed to 6x10^3 Jm^{-3}K^{-1} and 3.5x10^6 Jm^{-3}K^{-1}, respectively. This simulation result clearly indicates that a strong electron-lattice heat-reservoir coupling causes a fast relaxation of T_e.

Since the remanence signals in the simulation showed fast relaxation as the repeat number of the multilayer increased, it would be reasonable to assume that G_{el} is proportional to the repeat number of multilayers.

D. Effect of Lattice-specific heat, C_l

Figure S5(c) shows the effect of C_l on T_e while G_{el} and C_{e0} were fixed to 6x10^{17} Wm^{-3}K^{-1} and 6x10^3 Jm^{-3}K^{-2} , respectively. The initial maximum amplitudes were identical, but the amplitude beyond 20 ps was decreased as C_l increased.

Since the simulated remanence signals showed similar behavior, we could assume that C_l also is proportional to the repeat number of multilayers.

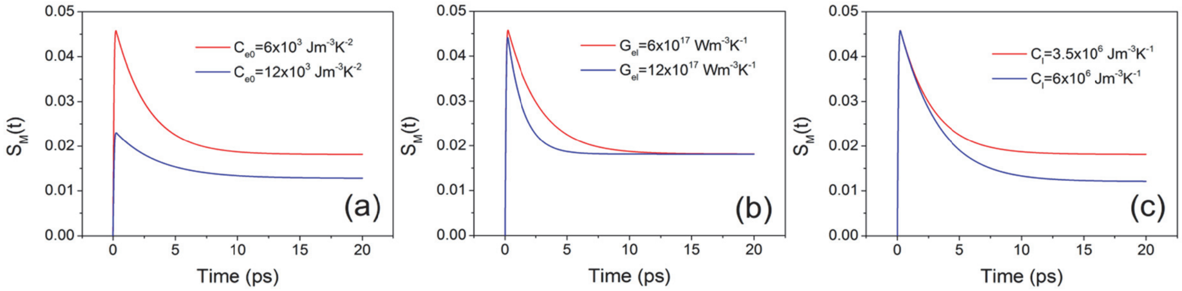

Figure S5. Numerical simulation results characterizing major effects of each of C_{e0}, G_{el}, and C_l. These results show that (a) the initial maximum amplitude of $S_M(t)$ decreases as C_{e0} increases, (b) a strong electron-lattice coupling causes a fast relaxation of T_e, and (c) as C_l increases, the initial maximum amplitude remains the same, but the amplitude beyond 10 ps decreases.

The discussion in the preceding section suggests that in order to globally fit the experimental signals for all of the multilayer samples according to their different repeat numbers, the following changes of various coefficients need to be made:

$$
\begin{aligned}
&C_e(t) = C_{e0} T_e(t) \\
&G_{el,i} = G_{el,1} + (i-1) \times \Delta G_{el}\ , \\
&C_{l,i} = C_{l,1} + (i-1) \times \Delta C_l
\end{aligned}
\qquad \text{S(5)}
$$

where i =1, 2, and 3 corresponds to the multilayer samples with 5, 10, and 15 bi-layers, respectively. C_l is assumed not to be a function of the lattice temperature, since it has only a weak temperature dependence at high temperatures.

We used the two-temperature model to globally fit all of the remanent magnetization signals with the constraints given in Eq. S(5). In all of the fittings, we also included the numerical convolution between $S_M(t)$ and a normalized Gaussian function, $G(t)$, to account for the pulse duration of the probe beam:

$$
f(t) = \int G(t-\tau) \cdot S_M(t) d\tau\ . \qquad \text{S(6)}
$$

All of the experimental remanence signals were fit using Eq. S(6).

References

[1] Wang, J. in *Optical techniques for solid-state materials characterization* Ch. 13, 467 (Taylor & Francis Group, LCC, 2012).

[2] Mizukami, S., Sajitha, E. P., Watanabe, D., Wu, F., Miyazaki, T., Naganuma, H., Oogane M. & Ando, Y. Gilbert damping in perpendicularly magnetized Pt/Co/Pt films investigated by all-optical pump-probe technique. *Appl. Phys. Lett.* **96**, 152502 (2010).

[3] Mizukami, S., Watanabe, D., Kubota, T., Zhang, X., Naganuma, H., Oogane, M., Ando, Y. & Miyazaki, T. Laser-induced fast magnetization precession and Gilbert damping for CoCrPt alloy thin films with perpendicular magnetic anisotropy. *Appl. Phys. Express* **3,** 123001 (2010).

[4] Capua A., Yang S., Phung T., and Parkin S. S. P. Determination of intrinsic damping of perpendicularly magnetized ultrathin films from time-resolved precessional magnetization measurements. *Phys. Rev. B* **92**, 224402 (2015).
